# Nanometer-Scale Pore Characteristics of Lacustrine Shale, Songliao Basin, NE China

**DOI:** 10.1371/journal.pone.0135252

**Published:** 2015-08-18

**Authors:** Min Wang, Jinxiu Yang, Zhiwei Wang, Shuangfang Lu

**Affiliations:** 1 Research Institute of Unconventional Petroleum and Renewable Energy (RIUP&RE), China University of Petroleum (East China), Qingdao, Shandong Province, 266580, China; 2 CSIRO Energy Flagship, 11 Julius Avenue, North Ryde, New South Wales 2113, Australia; Old Dominion Univ., UNITED STATES

## Abstract

In shale, liquid hydrocarbons are accumulated mainly in nanometer-scale pores or fractures, so the pore types and PSDs (pore size distributions) play a major role in the shale oil occurrence (free or absorbed state), amount of oil, and flow features. The pore types and PSDs of marine shale have been well studied; however, research on lacustrine shale is rare, especially for shale in the oil generation window, although lacustrine shale is deposited widely around the world. To investigate the relationship between nanometer-scale pores and oil occurrence in the lacustrine shale, 10 lacustrine shale core samples from Songliao Basin, NE China were analyzed. Analyses of these samples included geochemical measurements, SEM (scanning electron microscope) observations, low pressure CO_2_ and N_2_ adsorption, and high-pressure mercury injection experiments. Analysis results indicate that: (1) Pore types in the lacustrine shale include inter-matrix pores, intergranular pores, organic matter pores, and dissolution pores, and these pores are dominated by mesopores and micropores; (2) There is no apparent correlation between pore volumes and clay content, however, a weak negative correlation is present between total pore volume and carbonate content; (3) Pores in lacustrine shale are well developed when the organic matter maturity (Ro) is >1.0% and the pore volume is positively correlated with the TOC (total organic carbon) content. The statistical results suggest that oil in lacustrine shale mainly occurs in pores with diameters larger than 40 nm. However, more research is needed to determine whether this minimum pore diameter for oil occurrence in lacustrine shale is widely applicable.

## Introduction

The growing demand for energy has resulted in continuously increasing consumption of conventional oil and gas resources and has driven a new wave of exploration for oil and gas. In addition to exploration for conventional petroleum, unconventional oil and gas are now attracting more attention. In particular, the ‘shale gas revolution’ in North America has triggered a worldwide upsurge in shale gas exploration. More recently, an emphasis has been placed on shale oil exploration and development, stimulated by decreases in natural gas prices.

A good understanding of the shale reservoir, especially the shale oil storage mechanism, is of great importance to shale oil exploration and development, which necessitates the determination of pore type, size, and PSD. However, it is difficult to characterize the PSD of shale using conventional experimental and analytical methods, probably due to influencing factors, such as the small size of shale pores (nanometer-scale), wide range of pore sizes, maturity, TOC, and mineral contents, etc.

Recently, many researchers studied pore types and sizes of gas producing shales, using FIB-SEM (focused ion beam-scanning electron microscope), FE-SEM (field emission scanning electron microscope), CT scanning (micron and nanometer scale), gas adsorption (low pressure CO_2_ and N_2_ adsorption), and high pressure mercury injection methods. Some progress has been made toward understanding the controlling factors of gas content, shale microstructure, and gas flow mechanisms in marine shale [1–12]. However, as the shale oil/tight oil exploration work started fairly recently, international papers on the reservoir features of marine shales in the oil generation stage are quite limited [13–15], let alone the lacustrine shale. Two aspects of the difference between shale oil and shale gas are presented: (1) As molecular radius of oil is much larger than that of gas, which makes it quite difficult for oil to flow, the reservoir space in shale that is favorable to the accumulation of gas may not be necessarily effective for oil; (2) The microscopic pore structure of shale in the oil generation stage is different from gas shale, probably affected by the diagenesis and hydrocarbon generation processes.

In this article, 10 lacustrine shale core samples covering three maturity stages (immature, Ro < 0.5%; low-mature, 0.5% < Ro < 0.7%; mature, 0.7% < Ro < 1.3%) were analyzed to examine the pore shapes, types, PSDs, volumes, and their potential influence factors; which include TOC contents, maturities, and mineral compositions; by using the Rock-eval pyrolysis, XRD, SEM, and pore size distribution measurement techniques. Finally, the relationship between the pore diameter and the shale oil enrichment was investigated using a statistical method.

## Samples and Methods

### 2.1 Samples

**Fig 1:**
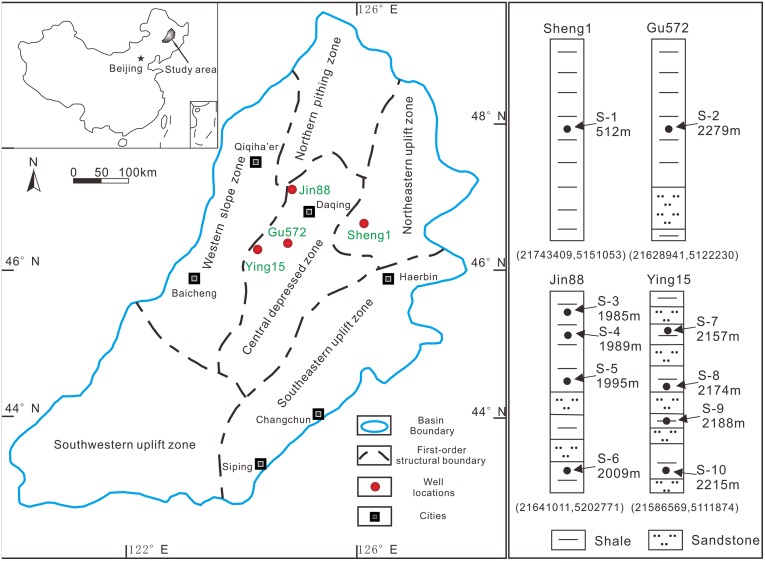
Geologic map of Songliao Basin, with well locations (red dots), sampling depths, and geographic coordinates indicated.

Lacustrine shale core samples taken from the first member of the Cretaceous Qingshankou Formation (K_2_qn^1^), in Suihua sag and Qijia Gulong sag in the north part of the Songliao Basin, NE China (Fig 1), were provided by the Daqing Oilfield, along with permission to use the samples for this publication. Shale of K_2_qn^1^ was deposited in a semi-deep lake during a global sea level rise [16] and is contemporaneous with a global Cenomanian/Turonian anoxic event [17,18]. Formations deposited during this event contain major oil-prone source rocks, which reach oil shale quality in their lower part (1^st^ member; K_2_qn^1^). The stratigraphic structure of Songliao Basin has been well studied [19,20].

### 2.2 Methods

#### 2.2.1 Geochemical and mineral analyses

TOC content was measured using the LECO CS230 apparatus, and combustion was performed using oxygen gas at temperatures between 350°C and 520°C. Rock-Eval analysis was performed on ~100 mg of crushed shale samples using a Rock-Eval 6. Free oil or volatile hydrocarbon content, expressed as mg HC/g rock (S_1_), the residual hydrocarbon generation potential, expressed as mg HC/g rock (S_2_), the temperature of maximum pyrolysis yield (T_max_), and the quantity of pyrolyzate, expressed as mg HC/g rock generated from kerogen during gradual heating in a helium stream are normalized to TOC to give the hydrogen index (HI; mg HC/g TOC). The pyrolysis stage (under an N_2_ environment) involved heating the sample at an initial iso-temperature of 300°C for 3 min to release the free hydrocarbons in the samples, followed by increasing the temperature to 650°C at a rate of 25°C/min to release the potential hydrocarbon, through thermal cracking. Random vitrinite reflectance (Ro%) measurement was performed on polished sections of the shale blocks using a Leitz MPV-3 photometer microscope, and the observation points are more than 20.

Shale samples were analyzed for mineral composition by XRD analysis, using a Panalytical X’Pert PRO Diffractometer with a Cu Ka radiation (40 kV, 30 mA) and scanning speed of 2o 2θ per minute. Crushed samples (80–100 mesh) were mixed with ethanol, hand ground in a mortar and pestle, and then ‘smear-mounted’ on glass slides for analysis. Prior to separation of the clay fraction (<2 μm), the samples were treated by adding 15% acetic acid solution to remove the soluble carbonates, in order to analyze the clay species. The excess acid was subsequently removed by multiple rinses with deionized water. The <2 μm clay fraction suspended on the top of the column was finally separated from the stabilized aqueous suspension using Stoke's Law. Oriented <2 μm clay fraction specimens were prepared by smearing the paste onto a glass slide, which minimizes size fractionation of the clay particles. For each sample, three X-ray analyses were performed: the first run after air-drying, the second run after ethylene—glycol solvation for 4 h at 80°C, and the third run after ethylene-glycol solvation for 2.5 h at 550°C. The clay mineral types were identified based on their characteristics in the X-ray diffraction patterns from the three XRD runs, following standard procedures as described in the Oil and Gas Industry Standard of the People's Republic of China [21].

#### 2.2.2 SEM

In the SEM experiment, 10 g samples were cut and polished to create fresh flat surfaces; which can then be fixed to a copper sample stage with latex, naturally dried for 72 hours, and the surface coated with a 2 nm thick galvanized gold film to enhance electric conductivity. After preparation, the samples were observed under VEGA/LMU scanning electron microscope from TESCAN Company. In the FE-SEM experiment, shale blocks weighing ~2–3 g were selected and packed with epoxy resin in order to guarantee the integrity of the crumbly shale samples. A 5 to 10 kV high-energy argon ion beam from a JEOL IB-09010 argon ion polishing instrument (made in Japan) was used to bombard the sample surface, which removed of the atoms of the bombarded surface layer by layer, generating a smooth and flat ion etched surface. After polishing, a 10 nm thick carbon film was sprayed on the ion etched surface to enhance the conductivity, which improved the image quality. After sample preparation, a high resolution field emission scanning electron microscope (HITACHI-S5500) was used to take electron images of the microscopic pores on the etched surfaces.

#### 2.2.3 Low pressure N_2_ and CO_2_ adsorption

Approximately 10 g of deoiled, dehydrated powders (less than 3 mm in size) were put into the sample tube, which was connected with a Quantachrome Nova 4200e apparatus. Each sample was degassed at 100°C for 8 h in a vacuum chamber prior to analysis to remove any residual volatile material. Then, the relevant experiment adsorbate (N_2_ or CO_2_) was injected into the sample tube. N_2_ adsorption isotherms were performed at 77 K with 20 points in a range of 0.01 < P/P_0_ < 0.985 with an equilibrium time of 120 s. A 30-point desorption isotherm was also performed from 0.2 < P/P_0_ < 0.985. Due to the wide range of relative pressures, this technique can be used to examine the micropores (<2 nm), mesopores (2–50 nm), and macropores (>50 nm). The surface area was calculated within a relative pressure range of 0.05–0.20 by the multi-point BET equation [22], pore volume and pore size distributions were calculated by the BJH (Barrett-Joyner-Halenda) method, using the adsorption branch of the N_2_ isotherms. This classical pore size model is based on the Kelvin equation assuming a cylindrical pore and corrected for multilayer adsorption [23].

Carbon dioxide adsorption isotherms were collected within a relative pressure range of 10^−5^–3.0 × 10^−2^ in an ice/water bath (273.15 K) with an equilibrium time of 90 s for each point. The PSDs up to 1.4 nm were determined from CO_2_ isotherms using the DFT (density functional theory) method, which can provide a more accurate approach to pore size analysis [24,25]. Micropore volume and surface area were calculated using the Dubinin-Radushkevich (D-R) equation [26,27].

#### 2.2.4 High pressure mercury injection method

First, the shale core samples were crushed to ~ 3 mm and deoiled, and then the samples were dehydrated at 110°C for 2 hours. After the pretreatment, the powders were put into a dilatometer with a volume of ~1 cm^3^; this work must be carried out in the glove-box under a nitrogen atmosphere to prevent the samples from being contaminated by the re-absorption of water vapor. Then, the dilatometer with samples was transferred to a porosimeter for vacuum degassing treatment under low pressure, after which liquid mercury was injected for pore detection under high pressure (~200 Mpa) by using a porosimeter (PoreMaster GT 60). Specific surface area and pore volume were calculated using the Young-Duper equation.

## Results

### 3.1 Geochemical characteristics and mineralogical compositions

The geochemical experimental results show that the samples have high TOC contents (see Table 1), with a minimum TOC content of 1.73 wt.%, a maximum of 4.21 wt.%, and an average of 2.72 wt.%. The hydrogen index (HI) ranges from 209 to 934 mg HC/g TOC; and the maturity of organic matter ranges from 0.43 to 1.28% (VR), spanning over three stages, which include immature, low maturity, and mature. The XRD analysis results show that the samples studied have a relatively high clay content, ranging from 59.6 to 65.9 wt.%. The clay content is dominated by a mixed-layer of illite and smectite (I/S), followed by quartz (17.2 to 25.0 wt.%), feldspar (7.6 to 13.6 wt.%), pyrite (0 to 4 wt.%), calcite (0.5 to 4.8 wt.%), and minor amounts of dolomite. The content of the illite and smectite mixed-layer is between 65% and 84%, ~75.7% on average; the content of illite is 9%-16%, ~11.3% on average; and kaolinite and chlorite only account for a small proportion (Table 2).

**Table 1:** Geological and geochemical parameters of studied samples.

Sample	Well	Depth	Ro	TOC	T_max_	S_1_	S_2_	HI	S_1_/TOC×100
Number		(m)	(%)	(wt.%)	(°C)	(mg HC/g rock)	(mg HC/g rock)	(mg HC/g TOC)	(mg HC/g TOC)
S-1	Sheng1	512	0.43	4.21	441	0.13	33.7	800	3.09
S-2	Gu572	2279	0.57	1.99	446	1.61	18.58	934	80.9
S-3	Jin88	1985	0.9	1.73	444	1.28	11.06	639	73.99
S-4	Jin88	1989	0.9	4.14	447	2.54	37.57	907	61.35
S-5	Jin88	1995	0.91	2.99	445	1.72	24.06	805	57.53
S-6	Jin88	2009	0.95	2.77	427	3.73	5.79	209	134.66
S-7	Ying15	2157	1.2	2.36	431	3.39	6.89	292	143.64
S-8	Ying15	2174	1.23	2.81	441	2.88	8.15	290	102.49
S-9	Ying15	2188	1.25	2.1	436	3.91	7.1	338	186.19
S-10	Ying15	2215	1.28	2.13	440	2.43	6.49	305	114.08

**Table 2:** XRD analysis data of studied samples.

Sample Number	Minerals(wt.%)						Clay components (%)			
	Quartz	Feldspar	Calcite	Dolomite	Pyrite	Clay	I/S	I	K	C
S-1	21.0	8.9	1.2	/	3.5	65.4	74	16	4	6
S-2	25.0	8.7	4.8	/	1.9	59.6	65	10	4	21
S-3	20.2	11.2	1.9	/	4.0	62.7	81	10	3	6
S-4	17.2	7.7	2.1	7.9	3.9	61.2	77	12	3	8
S-5	21.4	8.1	3.8	/	2.6	64.1	84	9	2	5
S-6	21.1	10.9	2.1	/	0.0	65.9	77	12	4	7
S-7	22.8	11.8	1.1	/	4.2	60.1	76	10	3	11
S-8	22.3	13.6	0.5	/	1.8	61.8	79	10	2	9
S-9	20.7	11.7	2.0	/	2.7	62.9	74	13	3	10
S-10	24.3	7.6	2.9	/	2.8	62.4	70	11	5	14

Note: I-Illite, K-Kaolinite, C-Chlorite, I/S- Illite/Smectite mixed layer.

### 3.2 Pore Types

**Fig 2:**
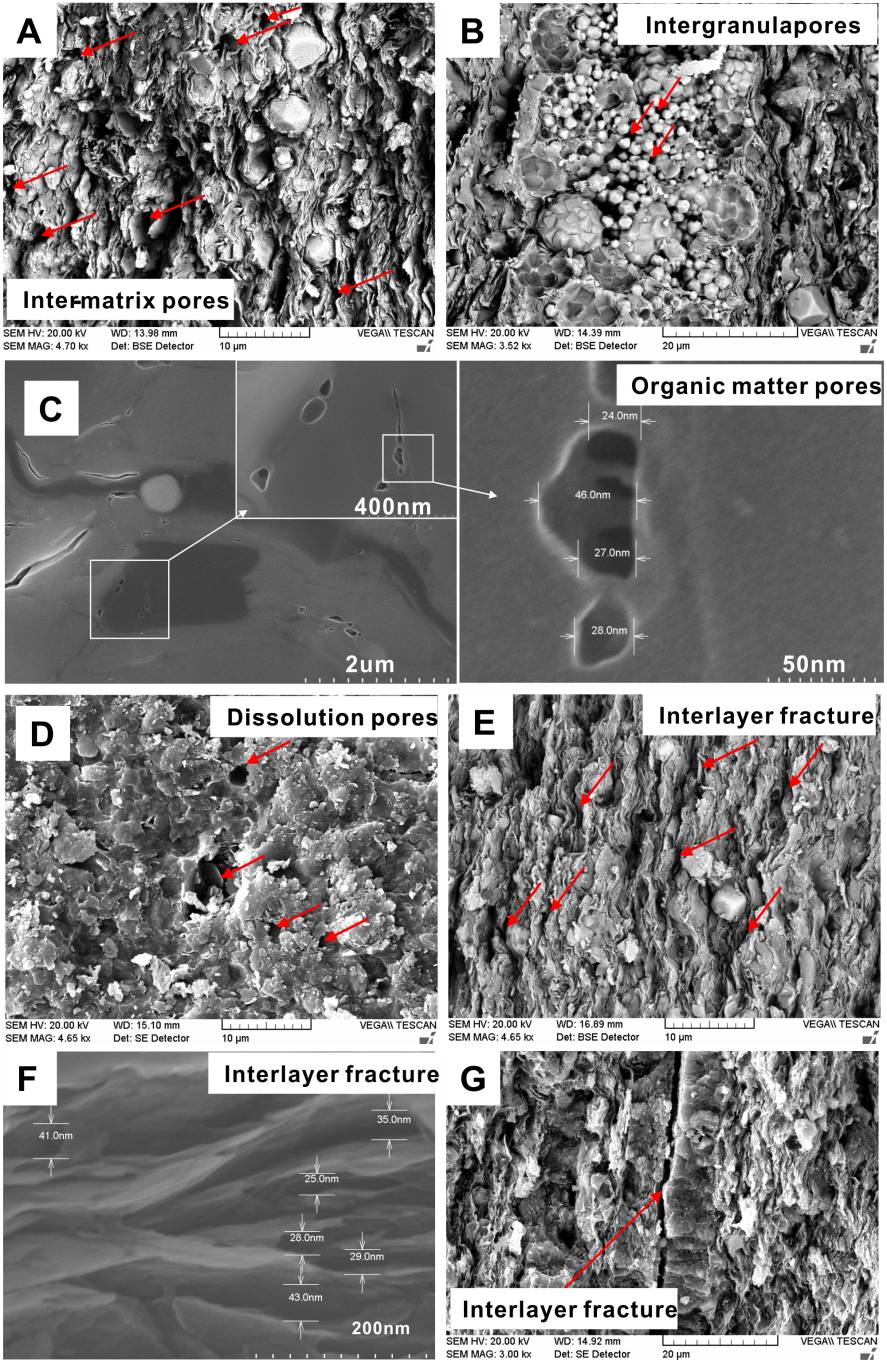
Pore and fracture characteristics of the studied samples. (A) Inter-matrix pores in sample S-2; (B) Intergranular pores in sample S-3; (C) Organic matter pores in sample S-5; (D) Dissolution pores in sample S-9; (E): Clay interlayer fractures in sample S-8; (F) Well-developed clay interlayer fractures in sample S-5; (G) Interlayer fractures between the calcite and clay layers in sample S-1.

According to the positions of the pores and the contact relations between the pores and minerals, pores types were identified in the studied samples. The pore types identified include inter-matrix pores, intergranular pores, organic matter pores, dissolution pores, and interlayer fractures (Fig 2). Inter-matrix pores are a common pore type in shale; they occur in clay mineral flakes, clay aggregates, cement crystals and large debris particles, and their widths can ranges from 0.5 to 4.0 um (Fig 2A). Intergranular pores are often present as framboidal pyrite intergranular pores (Fig 2B). Organic matter pores mainly refer to those inside the organic glomerate or the residual pores left after hydrocarbon generation. There are disputes over whether organic matter pores occurred or not in shale in the oil generation stage. Curits et al. (2012) reported that organic matter pores were not well developed in the oil generation stage, and even though organic matter pores formed, the effect of dissolution and compaction might lead to pore collapse due to poor particle support [13,14]. However, Reed et al. (2014) observed nano-scale organic matter pores in the Barnett shale in the oil generation stage, which were interpreted to be kerogen organic pores rather than pyrolysis asphalt organic pores [15]. Organic matter pores of 20–50 nm in size are also observed in the studied shale in the oil generation window (Fig 2C). Dissolution pores mainly form due to the enlargement of previously existing inter-matrix pores, by dissolution of carbonate, phosphate, or aluminosilicate minerals (Fig 2D). In addition, interlayer fractures are also well developed in the lacustrine shale; these include two types of fractures, i.e., the fractures between clay mineral interlayers (Fig 2E and 2F), and the fractures between calcite and clay mineral interlayers (Fig 2G).

### 3.3 Pore characteristics

Pore characteristics of shale can be investigated using fluid invasion and X-ray methods. Fluid invasion methods often include high pressure mercury injection and low pressure N_2_ and CO_2_ adsorption [28,29]. X-ray methods include small angle X-ray scattering (SAXS), small angle neutron scattering (SANS), and ultra-small angle neutron scattering (USANS) [3,4,30]. The X-ray scattering method is more widely used in coal and material researches [6]. It is difficult to effectively describe the pores by only one method, due to the presence of various shale pore types (organic and inorganic), very small pore throats (nano-scale), and a wide range of pore size (micron to nanometer scale). In this study, the CO_2_ adsorption isotherm is used to estimate micropore (0–2 nm) volume and PSD by using the D-R equation and the DFT method, respectively. The N_2_ adsorption isotherm at low relative pressure (<0.3) is used to estimate the micropore (<2 nm) PSD and volume in minerals; the high relative pressure (0.3–0.98) isotherm is used to estimate the mesopore (2–50 nm) PSD and volume using the BJH method; and the macropore (>50 nm) volume and PSD is estimated using the high pressure mercury intrusion data.

#### 3.3.1 Adsorption isotherms

**Fig 3:**
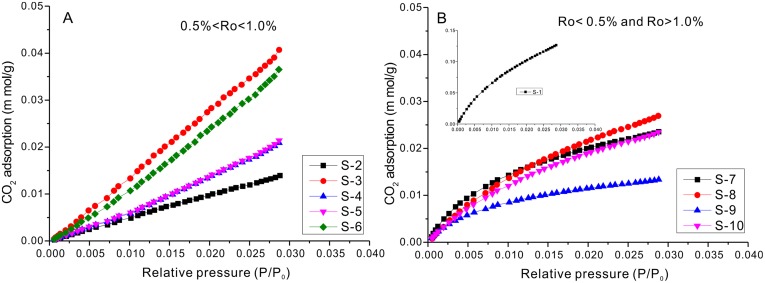
Carbon dioxide adsorption isotherms (273.15 K) for the studied samples.

**Fig 4:**
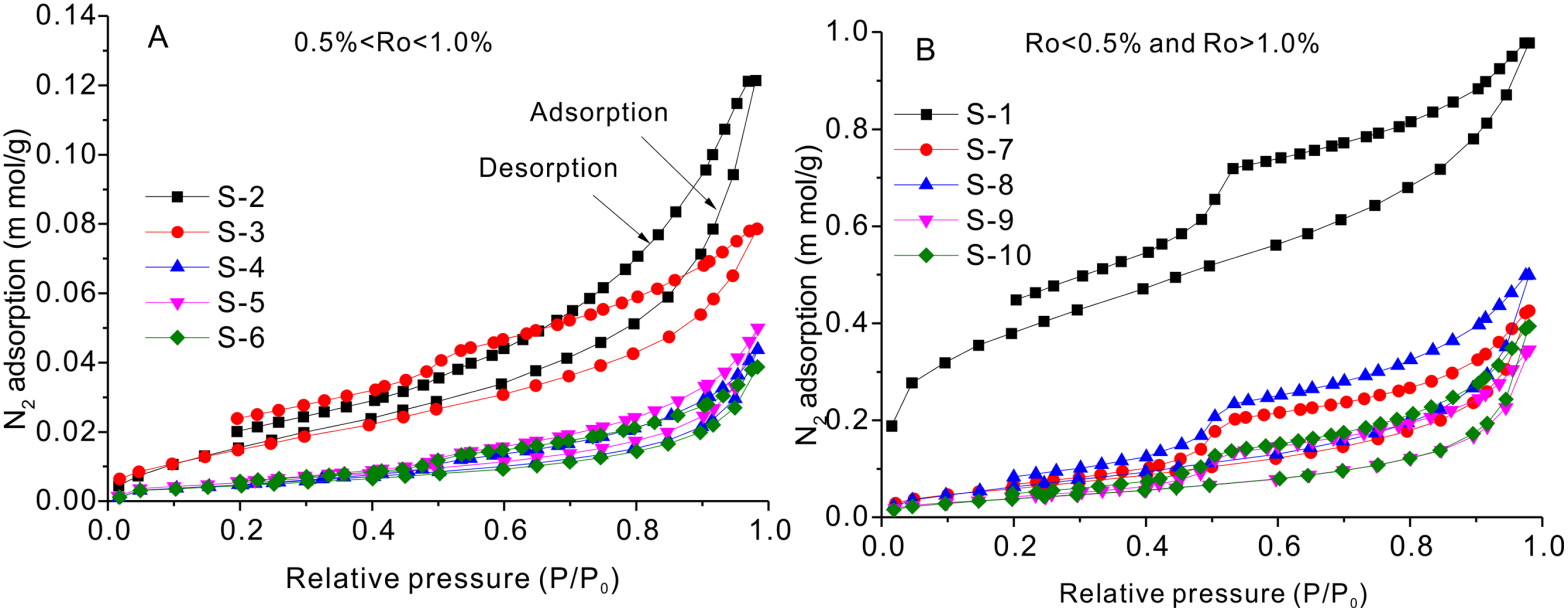
Nitrogen gas adsorption and desorption isotherms for the 10 shale samples at liquid nitrogen gas temperature (77.3 K).

**Fig 5:**
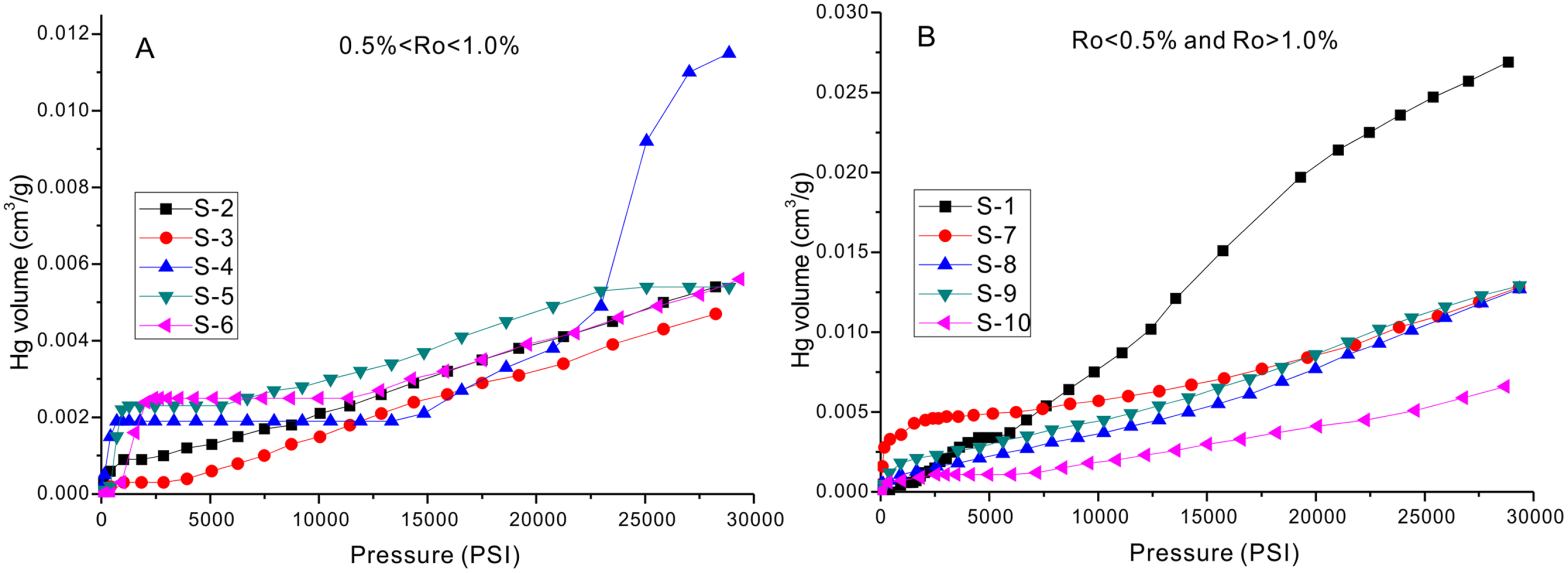
Hg intrusion volumes of the studied samples.

Pore parameters and adsorption isotherms from the above mentioned three experiments are shown in Table 3 and Figs 3–5. CO_2_ adsorption is coupled with the N_2_ adsorption analysis to examine the micropore size because the analytical temperature of N_2_ adsorption is too low (77.3 K) for the nitrogen molecules to access the micropores. The CO_2_-SA in the 10 samples ranges from 3.52 to 40.25 m^2^/g, with a mean value of 11.98 m^2^/g; and the N_2_-SA ranges from 0.39 to 30.99 m^2^/g, with an average of 5.17 m^2^/g (Table 3), which is lower than that of the CO_2_-SA. The surface area derived from N_2_ adsorption might be underestimated due to the activated diffusion and molecular sieving phenomenon [27,31,32]. The CO_2_ adsorption amount at a P/P_0_ value of ~0.03 varies from 0.0134 to 0.1265 m mol/g for different samples, and CO_2_ adsorption isotherms manifest as type I, which is indicative of microporous solids (Fig 3). The isotherms for the samples with maturity 0.5% < Ro < 1.0% (Fig 3A) show a linear curve, indicating a high micropore volume (Table 3) and a relatively larger pore diameter; however, CO_2_ isotherms of other samples (Fig 3B) exhibit a convex-upward curve, suggesting the coexistence of micropores and mesopores.

**Table 3:** Pore volumes and surface areas of lacustrine shale samples.

Sample number	TOC	CO_2_-SA	CO_2_-PV	N_2_-SA	N_2_-PV_0.3_	N_2_-PV_0.85_	N_2_-PV_0.98_	Hg-PV_50_	Hg-PV_7_
	wt.%	m^2^/g	cm^3^/g	m^2^/g	cm^3^/g	cm^3^/g	cm^3^/g	cm^3^/g	cm^3^/g
S-1	4.21	40.25	0.013	30.99	0.0149	0.0251	0.0339	0.0031	0.0269
S-2	1.99	5.40	0.002	1.49	0.0007	0.0021	0.0042	0.0012	0.0054
S-3	1.73	16.80	0.006	1.31	0.0007	0.0016	0.0027	0.0004	0.0047
S-4	4.14	7.96	0.003	0.39	0.0002	0.0006	0.0015	0.0019	0.0115
S-5	2.99	7.85	0.003	0.44	0.0002	0.0007	0.0017	0.0023	0.0054
S-6	2.77	14.97	0.005	0.39	0.0002	0.0006	0.0013	0.0025	0.0056
S-7	2.36	6.59	0.002	5.00	0.0025	0.0070	0.0148	0.0048	0.0128
S-8	2.81	9.02	0.003	5.14	0.0027	0.0078	0.0173	0.0021	0.0127
S-9	2.10	3.52	0.001	3.26	0.0016	0.0048	0.0119	0.0028	0.0129
S-10	2.13	7.43	0.002	3.32	0.0016	0.0049	0.0137	0.0011	0.0066

Note, SA- surface area; N_2_-PV_0.3_—pore volume derived from N_2_ adsorption isotherm at P/P_0_ <0.3; Hg-PV_50_—volume of pores with diameter higher than 50 nm, derived from high pressure mercury intrusion experiment.

The isotherms of N_2_ adsorption and desorption at the temperature of liquid nitrogen (-196°C) are shown in Fig 4. According to the IUPAC classification, the N_2_ adsorption isotherms belong to type IV [33], due to the presence of obvious hysteresis loops in all samples, and adsorption amounts ranging from 0.0388 to 0.9781 m mol/g for different samples for a P/P_0_ value of approximately 0.980 (Fig 4). The hysteresis loop is closed in most shale samples with only a few samples having open hysteresis loops (S-1, S-2, and S-3). The “forced closure” of the desorption branch at P/P0 ≈ 0.45 is interpreted to be the result of the Tensile Strength Effect, which is probably caused by the instability of a hemispherical meniscus during desorption in pores with critical diameters of approximately 4 nm [34]. The presence of hysteresis indicates that evaporation from pores is a different process from condensation within the pores and that capillary condensation occurs within the mesopores [35]. The lack of closure of the hysteresis loop below a relative pressure of 0.45 is interpreted to be caused by swelling or adsorption of nitrogen in the micropores [35], which has also been observed in coal and marine shale [6,7,36].

The shape of the hysteresis loop was complex for the studied samples (Fig 4), which is probably the result of the presence of a combination of several typical pore types (Fig 2). Close examination of the hysteresis loops shows two types, type H2 (S-1, S-3, and S-6 –S-10) and type H3 (S-2, S-4, and S-5) (Fig 4). The type H2 loop is interpreted to most probably be caused by the difference between the condensation and evaporation processes, both occurring in pores with narrow necks and wide bodies like ink-bottle pores. The type H3 hysteresis occurs in aggregates of plate-like particles, which have slit-shaped pores. The recognition of type H3 hysteresis loops must be done with caution because it is very susceptible to error [37]. This caution was echoed by Clarkson et al. (2012), who studied tight gas sandstones using USANS/SANS and gas adsorption analysis and found that the assumption of slit-shape pores inferred from the hysteresis loop shape was not consistent with the SANS scattering results[3]. However, slit-shaped pores have been observed in shale, such as the intraplatelet pores within plate-like clay aggregates [38], which is consistent with the development of clay mineral interlayer fractures observed under SEM (Fig 2E and 2F).

**Fig 6:**
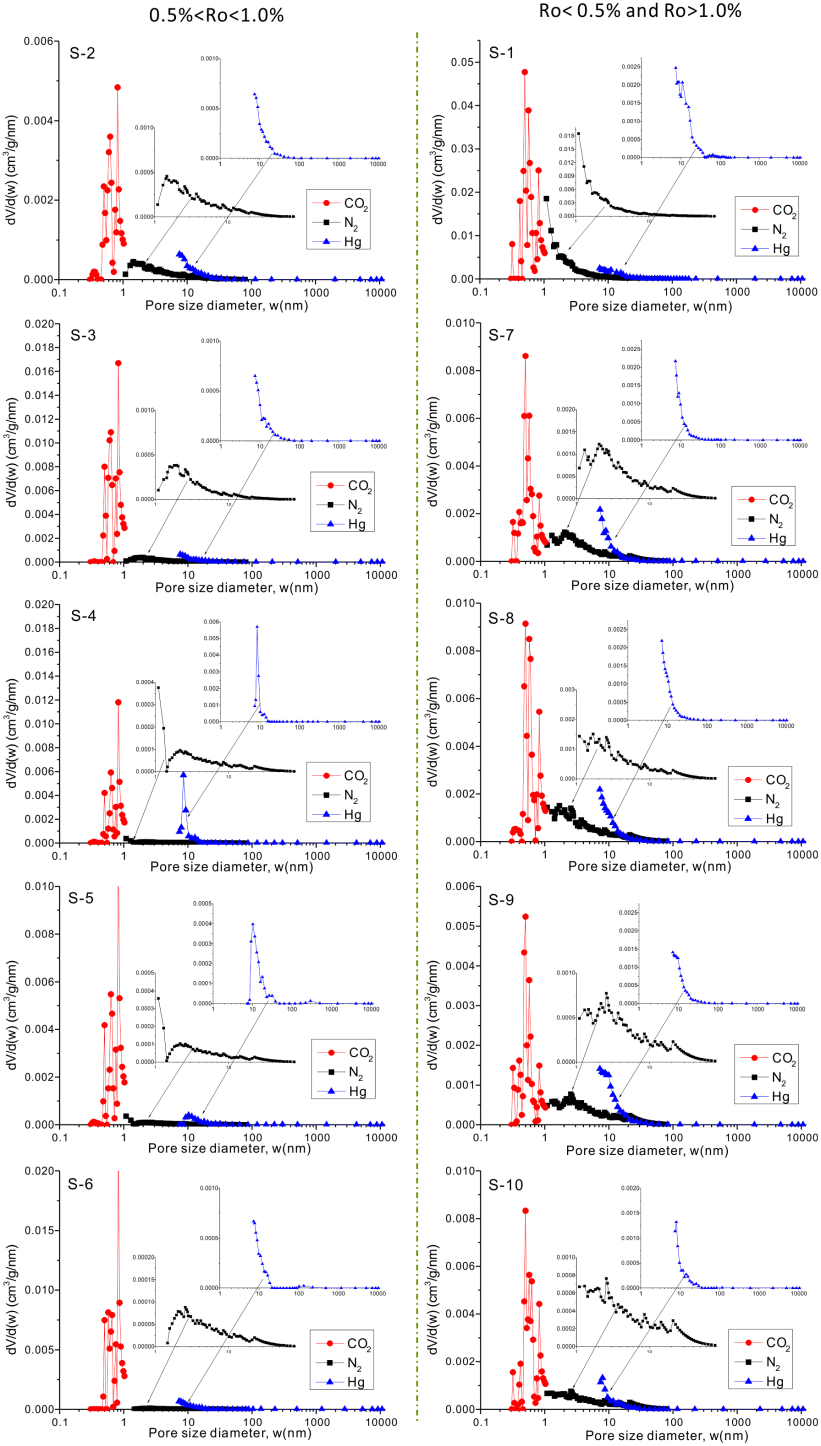
Combination of PSDs from CO_2_ (DFT model), N_2_ adsorption (BJH model), and Hg intrusion (Young-Duper equation) experiments for the studied samples.

Hg intrusion curves for the 10 samples are observed to be of two types. Samples S-4, S-5, S-6, and S-7 comprise one type, which exhibits a sharp increase in the lower pressure section, followed by a plateau, and finally a slow raise in the higher pressure condition (Fig 5). Sample S-4 is the exception to the last stage, as it shows a very quick increase in the very high pressure stage, which might be caused by the artificial fracture. The inconsistency of pore size distributions (< 50 nm) for N_2_ and Hg analyses also points to the artificial fracture of Sample S-4 (Fig 6 S-4). Samples S-1, S-2, S-3, S-8, S-9, and S-10 comprise a second type, in which the Hg intrusion curves increase slowly with increasing pressure (Fig 5).

#### 3.3.2 Pore size distribution (PSD)

**Fig 7:**
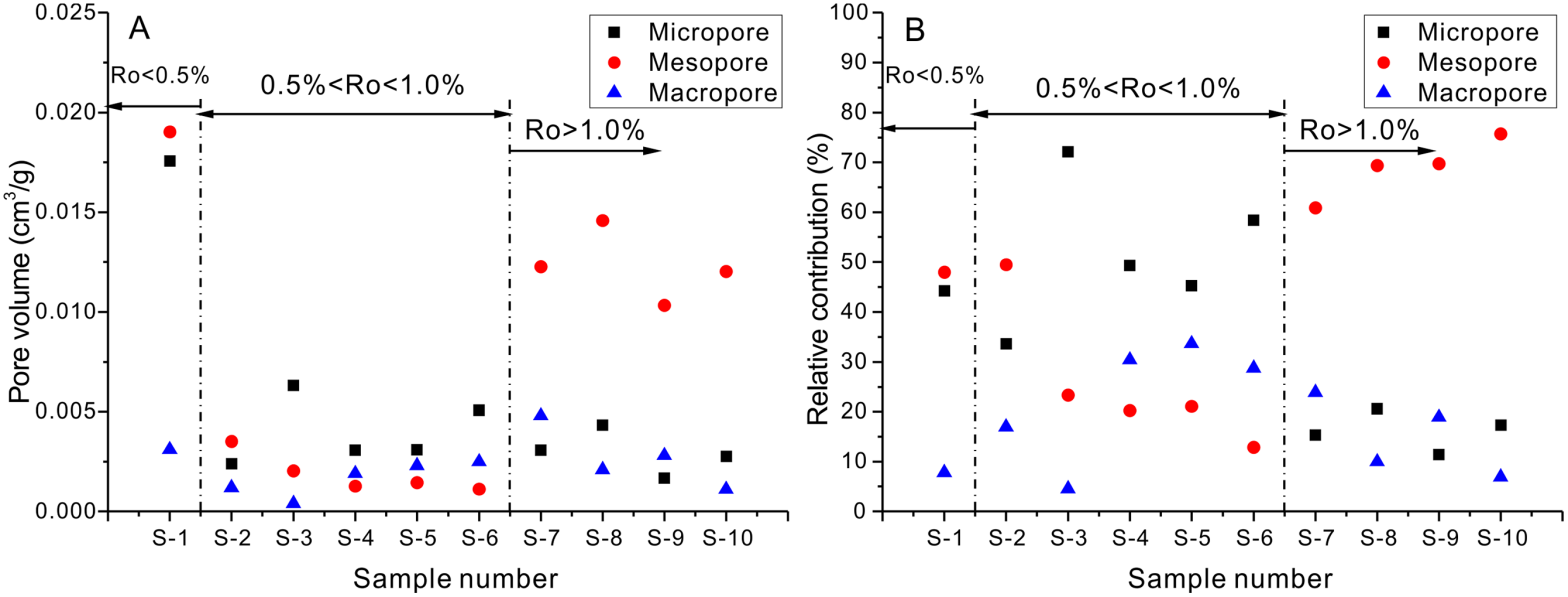
(A) Relationship between pore volumes and organic matter maturities; (B) Relationship between relative contributions of pore volume and organic matter maturities. Micropore volume = CO_2_-PV + N_2_-PV_0.3_ - N_2_-PV_0.1_; Mesopore volume = N_2_-PV_0.98_ - N_2_-PV_0.3_; Macropore volume = Hg-PV_50_.

The PSDs of the studied samples, which combine the results of N_2_ and CO_2_ adsorption and Hg intrusion analyses, are shown in Fig 6. The micropore size derived from the CO_2_ adsorption isotherm of S-1 (Ro < 0.5%) is similar to shales with maturity Ro > 1.0% (Fig 6, right column), which might be due to the sample’s lower compaction and diagenesis effects. Since shale oil usually occurs in the oil generation stage, the pore characteristics of immature shale will not be discussed further in this paper. For shales with an Ro value between 0.5% and 1.0% (Fig 6, left column), the micropores are 0.45–1.0 nm in diameter, most of which are 0.8–1.0 nm. For shales with maturity Ro > 1.0%, their micropores are 0.3–1.0 nm in diameter, among which most are 0.45–0.7 nm in diameter, followed by pores of 0.8–1.0 nm in diameter (Fig 6 S-7, 6 S-8, 6 S-9, and 6 S-10).

Most of the PSDs derived from N_2_ adsorption isotherms show a main peak at ~2 nm (Fig 6). For samples with a maturity lower than 1.0% (Ro), pore diameters range from 1 to ~ 40 nm (Fig 6 S-2, 6 S-3, 6 S-4, 6 S-5, and 6 S-6), with the exception of sample S-1, for which the pore diameter is smaller than ~10 nm (Fig 6 S-1). However, the pores in the high maturity (Ro > 1.0%) shales are relatively larger, with diameter that can reach as high as ~70 nm (Fig 6 S-7, 6 S-8, 6 S-9, and 6 S-10).

The incremental intrusion plots using Hg intrusion show pore volumes in the meso- and macropore range, and indicate that the macropores are not well-developed in all the studied samples (Fig 6). However, samples S-1, S-5, and S-6 still show some macropores with diameters ~70 nm, 300 nm, and 150 nm, respectively (Fig 6 S-1, S-5, and S-6). It is worth noting that most samples exhibit plentiful pores with diameters smaller than 10 nm, and most of those indicated by Hg intrusion are artificial enlarged due to the very high pressure, which can also be proven by the inconsistency between the PSDs derived from N_2_ adsorption isotherms and those derived from Hg intrusion (Fig 6).

Although previous studies indicated that the PSD curves from CO_2_ and N_2_ adsorption and mercury intrusion capillary pressure showed good correlations [4,5,25], when in theory, CO_2_ and N_2_ adsorption and mercury intrusion analyses should not yield similar results, except for a sample with very similar pore bodies and pore throats, which is impossible in shale. On the other hand, the different pretreatment methods also have some influence on the measured results (Wang, 2015, private communication). Therefore, the process of combining PSDs of shale pores from different measurement methods is still a challenge, though we have achieved preliminary results in this study (Fig 6).

## Discussion

### 4.1 Controlling factors of pore development

The experimental results show that the pore volumes obtained by using different testing methods have different relationships to maturities (Fig 7). The mesopore volume for samples with maturity Ro > 1.0% is obviously higher than for samples with maturity of <1.0% (excluding sample 1, Fig 7A). However, there is no apparent relationship between micropore and macropore volume and maturity (Fig 7A). The relative contribution of mesopore volume decreases first and then increases as the maturity increasing (Fig 7B); while relative contributions of micropore and macropore volume increase first and then decrease with increasing maturity (Fig 7B). All of the kinds of pores might not be well-developed in shale in the pre-oil generation stage (Ro < 1.0%); however, mesopores are well-developed in shale with Ro > 1.0%.

**Fig 8:**
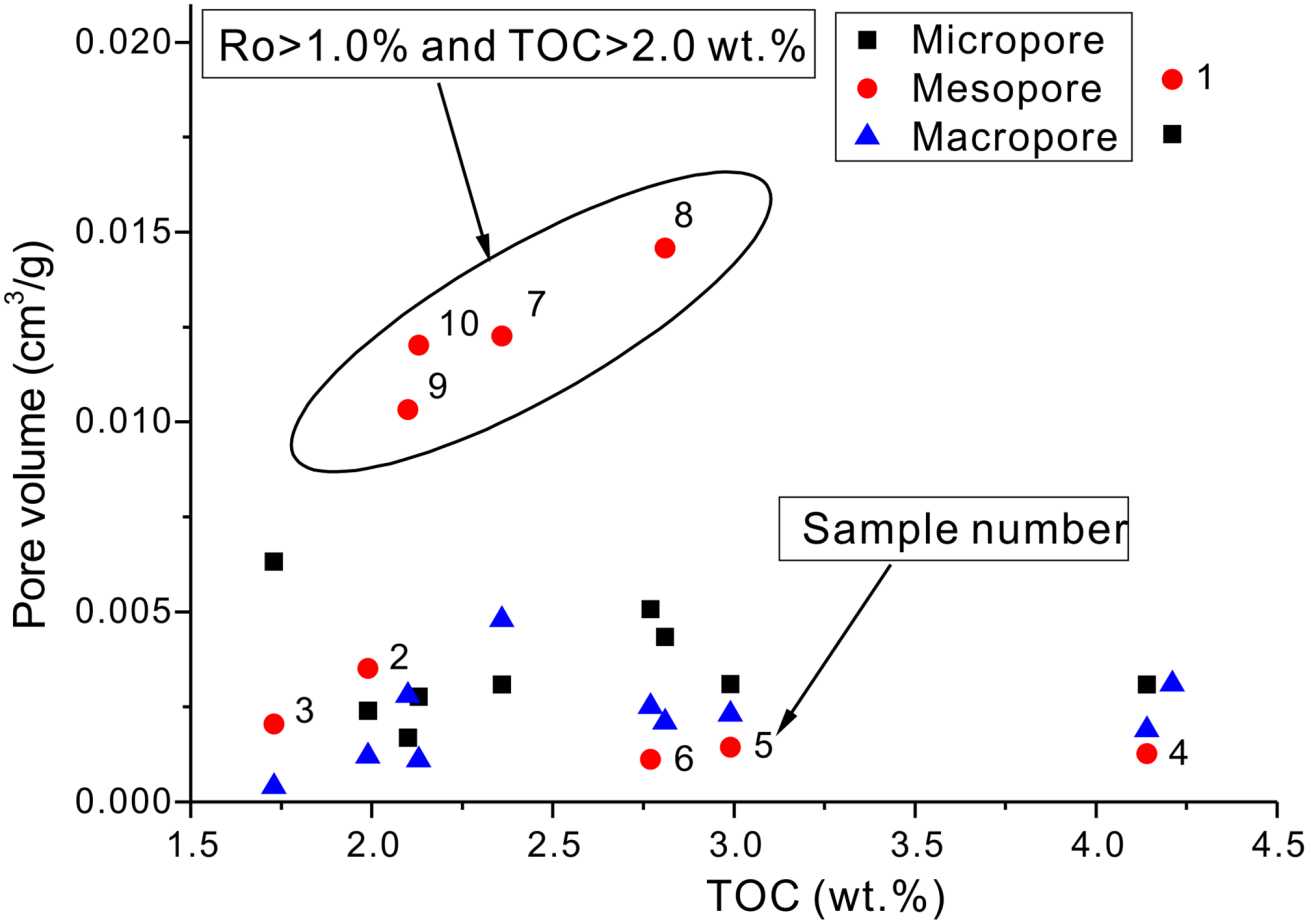
Relationship between TOC contents and pore volumes for the studied samples.

According to previous studies, the TOC content is often in positive correlation with total porosity; this positive correlation also exists between the TOC content and micropore or mesopore volumes [4,29,39,40]. However, Mastalerz et al. (2013) stated that there was no obvious positive or negative relationship between the TOC content and total porosity (their Fig. 11)[7]. In this paper, our observations show that there is a positive relationship between TOC values and pore volumes when the maturity, Ro, was >1.0% (Fig 8).

The relationship between pore volumes and TOC contents for samples with Ro > 1.0% shows that the pore volume of mesopores increases with increasing TOC contents (samples 7–10 in Fig 8); however, micropore and mesopore volumes show no apparent correlation with TOC contents (Fig 8). For shale samples with Ro < 1.0%, pore volumes have no apparent relationship with TOC contents either (Fig 8). Micropore volumes have no apparent correlation with TOC values, probably due to the organic matter pores being less developed. This less developed state can be ascribed to two reasons; the first is the lower expulsion efficiency of oil from shale resulting in the plugging of organic matter pores by residual hydrocarbons; and the second is that organic matter pores are not the primary pores for shale in the pre-oil generation stage (Ro < 1.0%). All these observations suggest that organic matter pores, mainly mesopores, are well-developed in the post-oil generation stage, probably due to the high expulsion efficiency and oil cracking. Although shale samples with Ro < 1.0% have also entered the oil window, organic matter pores are not well-developed.

**Fig 9:**
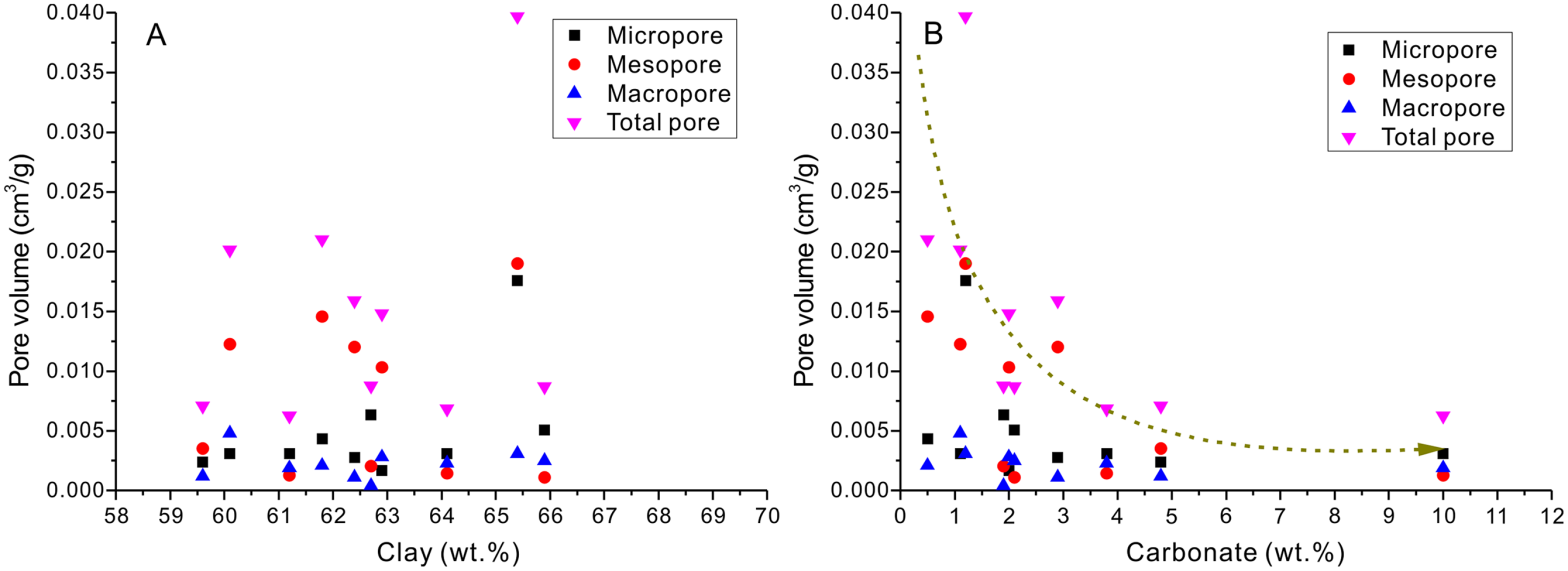
Relationship between pore volumes and mineral contents of the studied samples.

Mineral compositions and contents are other factors that may affect porosity and complicate the understanding of porosity evolution. Previous studies show that organic matter and clay minerals (dominantly illite) jointly contribute to the micropore volume in shale [5, 41–43]. Total porosity will increase with higher clay and/or quartz content and will decrease with higher carbonate content. However, due to the strong effect of maturity on total porosity, the influence of changes in mineralogical composition is often masked [7]. In this study, the experimental results show that the pore volume is not correlated with clay content (Fig 9A), which is probably due to the very small difference in clay content among these samples (from ~60 to 66 wt.%). The experimental results also show that there is a weak negative relationship between total pore volume and carbonate contents (Fig 9B).

### 4.2 Space of oil occurrence

In the past, researchers mainly focused on the features of storage space in shale reservoirs, with little research done on the relationship of storage space to shale oil enrichment. This is partly because the state of shale oil occurrence in pores is complex, making it difficult to observe and describe. For instance, oil adsorbed to organic matter or minerals has no distinct phase from the free oil in pores; and sample preparation before FIB-SEM analysis could also affect the quantity and position of shale oil occurrence. There are some methods to measure the space of oil/gas occurrence in fine-grained rocks. For example, Zou et al. (2011) estimated the critical pore radii (minimum radius) for tight gas and oil reservoirs in the Ordos Basin of China, which might be 40 nm and 54 nm, respectively, which is equal to the thickness of the irreducible water film plus the molecule diameter of methane or oil [44]. Therefore, only when pore radii are larger than the above values, can oil and gas flow in shale. Compared to tight sand, the oil-rock interaction in shale is much more complex, making the thickness of adsorbed oil film/bound oil film more difficult to estimate. Some scholars proposed a molecular dynamic method to simulate the adsorption and interaction between gas/oil and mineral texture [45–47]. But only simplified oil molecules and rock textures can be adopted in simulation because the oil molecules (e.g. the physical properties of crude oil) and the rock textures are very complex, which inhibiting a wide application of the obtained results. Other scholars directly observed oil occurrence in tight sand under E-SEM (environment scanning electron microscope) [48,49]; however, whether oil enrichment in shale can be observed under electron microscope or not is still a question. Apart from the theoretical consideration mentioned above, there are a few other methods, which may help determine the main range of pore size for oil storage, such as numerical simulation, observation under a microscope (E-SEM), and correlation between the oil content and pore volume.

**Fig 10:**
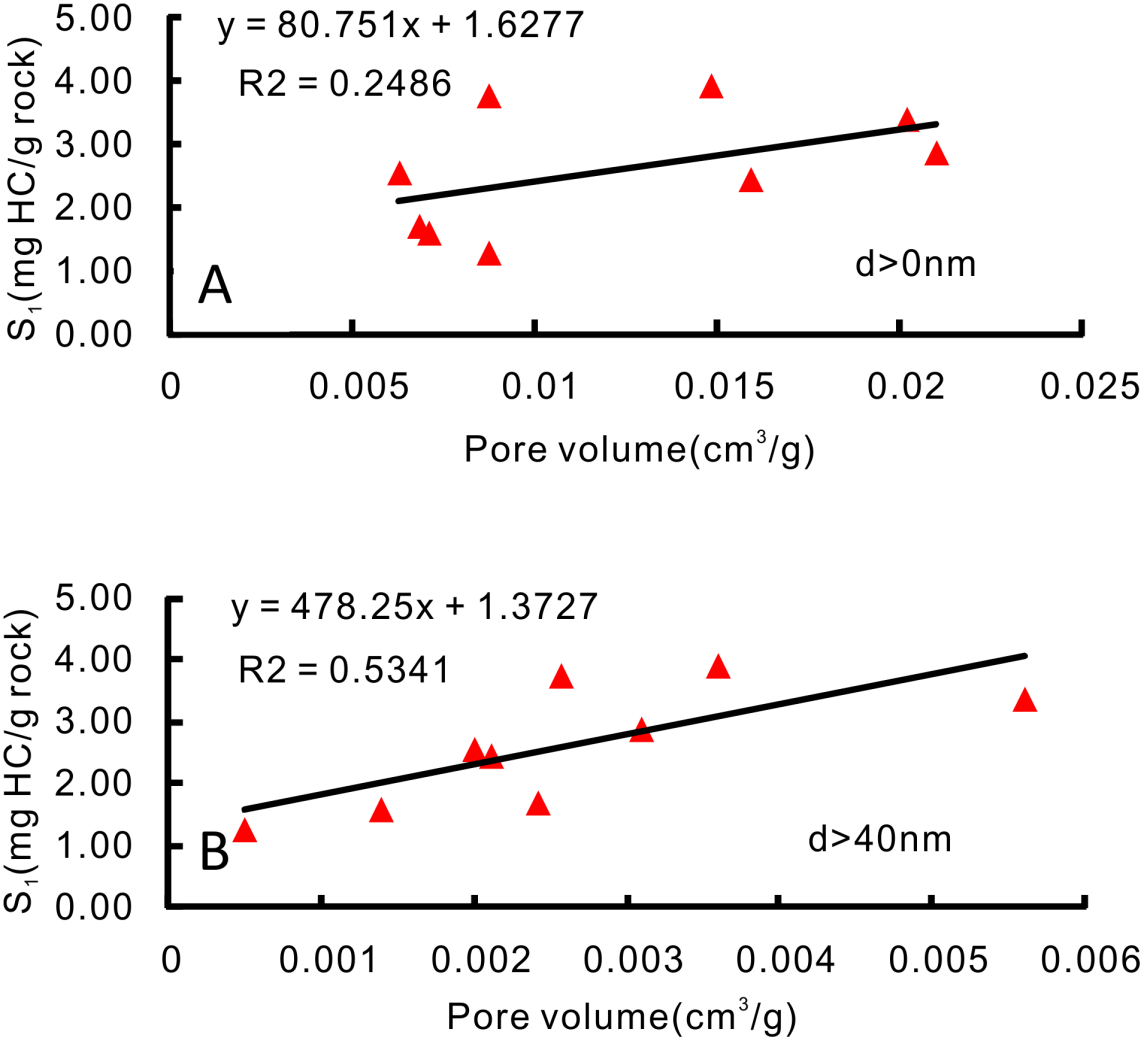
A—Total pore volume vs. S_1_; B—Volume of pores with diameter > 40 nm vs. S_1_.

In this article, we use a statistic method to infer the possible pores sizes or minimum diameter of pores for oil accumulation in shale. Commonly, there should be a linear relationship between the oil content and the pore volume if the pore is completely filled with oil or highly saturated with oil. Due to the very small pores in shale and relatively larger molecular diameter of oil, not all the pores are effectively filled with oil. Therefore, if oil was stored in pores with diameters higher than a special value, a positive linear relationship between the oil content and the volume of pores with diameter higher than the special value would be present (Fig 10). Based on the above assumed conditions, low pressure CO_2_ and N_2_ adsorption, and Hg intrusion experimental data, pore volumes for different pore sizes can be obtained, along with the correlation coefficients (R^2^) of the oil contents (S_1_) and the pore volumes (Table 4). The statistical results show that the correlation coefficients gradually increase with increasing D_c_ (the minimum diameter of pores that might be effective for oil charging); and when D_c_ reaches 40 nm, the correlation coefficient (r^2^) reaches its highest value (r^2^ is 0.534, Sig. F is 0.025, and P-values are lower than 0.05; Table 4). These results suggest the shale oil accumulation space has a significant positive relationship with the pores (diameter > 40 nm), and shale oil might accumulate in pores bigger in size than this critical value (40 nm), which account for 22% of the total pores. However, all the correlation coefficients (r^2^) are lower than 0.55, which quite possibly indicates pore diameter is not the sole parameter that has influence on shale oil occurrence; the viscosity, temperature, and pressure might also affect oil accumulation. This study’s conclusion is based on only 9 samples; therefore, the minimum diameter of pores for shale oil storage needs to be investigated further in more detail, using both theoretical calculations and observation under a microscope, and other potential influence factors should also be considered in future studies.

**Table 4:** The relationship of shale oil content (S_1_) and pore volumes for different pore sizes which are derived from CO_2_ adsorption, N_2_ adsorption, and Hg intrusion analyses.

Sample number	Oil content	Volume (100 x cm^3^) of pores with diameter > D_c_ (nm), D_c_ =									
	S_1_ (mg HC/g rock)	0	2	10	15	20	25	30	35	40	50
S-2	1.61	0.71	0.47	0.31	0.26	0.21	0.18	0.16	0.15	0.14	0.12
S-3	1.28	0.88	0.24	0.13	0.1	0.08	0.07	0.06	0.05	0.05	0.04
S-4	2.54	0.62	0.32	0.27	0.25	0.24	0.22	0.21	0.2	0.2	0.19
S-5	1.72	0.68	0.37	0.32	0.3	0.28	0.27	0.26	0.25	0.24	0.23
S-6	3.73	0.87	0.36	0.32	0.3	0.29	0.28	0.27	0.26	0.26	0.25
S-7	3.39	2.01	1.71	1.18	1.02	0.89	0.75	0.67	0.6	0.56	0.48
S-8	2.88	2.1	1.67	1.07	0.88	0.71	0.55	0.44	0.36	0.31	0.21
S-9	3.91	1.48	1.31	0.93	0.8	0.68	0.55	0.46	0.4	0.36	0.28
S-10	2.43	1.59	1.31	0.92	0.74	0.62	0.45	0.34	0.26	0.21	0.11
Correlation coefficients (r^2^)		0.249	0.281	0.342	0.385	0.421	0.475	0.514	0.533	0.534	0.48
Sig. F		0.172	0.142	0.098	0.075	0.059	0.04	0.03	0.026	0.025	0.039
P-value (Intercept)		0.172	0.142	0.098	0.075	0.06	0.04	0.03	0.026	0.025	0.039
P-value (X Variable)		0.056	0.007	0.01	0.012	0.014	0.02	0.025	0.027	0.027	0.02
Average proportion (%)		100	64	46	40	35	30	27	24	22	19

Note: the unit of S_1_ is mg HC/g TOC; D_c_ is the assumed minimum diameter of pores in which oil can be stored; r^2^ is the correlation coefficient of the linear fitting equation for pore volumes and S_1_ values; and average proportion is the average ratio of pore volumes (diameter > d_c_) to the total pore volume for the studied samples.

## Conclusions

1. The pore types developed in lacustrine shale include inter-matrix pores, intergranular pores, organic pores, dissolution pores, and interlayer fractures (e.g. clay mineral interlayers or calcite and clay mineral interlayers). Most pores are mesopores or micropores and are less than 50 nm in diameter.
2. Nano-scaled pores are well-developed in shale with Ro > 1.0%, and are positively correlated with TOC contents. However, when the organic matter maturity is <1.0%, pores are poorly developed, and the pore volume is not correlated with maturity or TOC content. In addition, the degree of development of the pores shows no obvious relationship with clay and carbonate contents.
3. Statistics on S_1_ contents and pore volumes with different pore sizes show that shale oil mainly exists in pores with diameter larger than 40 nm.
